# Picosecond Laser-Induced Hierarchical Periodic Near- and Deep-Subwavelength Ripples on Stainless-Steel Surfaces

**DOI:** 10.3390/nano10010062

**Published:** 2019-12-26

**Authors:** Shijie Ding, Dehua Zhu, Wei Xue, Wenwen Liu, Yu Cao

**Affiliations:** 1 College of Mechanical & Electrical Engineering, Wenzhou University, Wenzhou 325035, Chinaxw@wzu.edu.cn (W.X.); sophialww@163.com (W.L.); 2 Institute of Laser and Optoelectronic Intelligent Manufacturing, Wenzhou University, Wenzhou 325035, China; zhu_556@163.com

**Keywords:** laser induced subwavelength ripples, surface plasmon polaritons, coulomb explosion, stainless steel

## Abstract

Ultrafast laser-induced periodic surface subwavelength ripples, categorized based on the ripple period into near-subwavelength ripples (NSRs) and deep-subwavelength ripples (DSRs), are increasingly found in the variety of materials such as metals, semiconductors and dielectrics. The fabrication of hierarchical periodic NSRs and DSRs on the same laser-irradiated area is still a challenge since the connection between the two remains a puzzle. Here we present an experimental study of linearly polarized picosecond laser-induced hierarchical periodic NSRs and DSRs on stainless-steel surfaces. While experiencing peak power density higher than a threshold value of 91.9 GW/cm^2^, in the laser-scanned area appear the hierarchical periodic NSRs and DSRs (in particular, the DSRs are vertically located in the valley of parallel NSRs). A large area of the uniformly hierarchical periodic NSRs and DSRs, with the spatial periods 356 ± 17 nm and 58 ± 15 nm, respectively, is fabricated by a set of optimized laser-scanning parameters. A qualitative explanation based on the surface plasmon polariton (SPP) modulated periodic coulomb explosion is proposed for unified interpretation of the formation mechanism of hierarchical periodic NSRs and DSRs, which includes lattice orientation of grains as a factor at low peak power density, so that the initial DSRs formed have a clear conformance with the metallic grains.

## 1. Introduction

The laser-induced periodic surface structure (LIPSS) [1,2], a by-product effect of laser material processing applications is at its inception, but in the past few years has been a research hotspot that involves multiple branches of physics such as wave optics, non-linear optics, fluid dynamics and thermodynamics. With the rapid development in particular of ultrafast laser-matter interaction in both science and engineering, the ultrafast laser-induced periodic surface subwavelength ripples [3,4,5] which can be categorized based on the ripple period (Λ) into near-subwavelength ripples (NSRs, 0.4 < Λ/λ < 1) and deep-subwavelength ripples (DSRs, Λ/λ < 0.4) [3], have been reported increasingly on a variety of materials such as metals [5,6], semiconductors [7,8] and dielectrics [3,9].

However, the existing formation mechanisms for construction of NSRs and DSRs are considered completely different, and their theoretical models have been in a state of debate and development [3,10,11]. The NSRs (sometimes called classical ripples) are found to be formed by a wide range of pulse durations from a few hundred nanosecond (ns) to femtosecond (fs) laser irradiation on the solid material surface, which makes it seem like a universal pulse laser-induced material response. The orientation of NSRs, which were found always parallel or perpendicular to the incident laser polarization, is usually ascribed to the periodic electric field modulation by the incident laser beam interference with surface scattering laser wave due to the sample surface roughness [12,13]. The excitation of surface plasmon polaritons (SPPs) by incident laser pulses is also widely used for the explanation of NSR formation [3]. Huang et al. [3] compared experiments with metal, semiconductor and dielectric and assumed the grating-assisted surface plasmon (SP)-laser coupling should be responsible for the origination of NSRs. Reif et al. [14,15] considered the ion sputtering and thin liquid film, and proposed the self-organized effect of the non-stable material to explain the formation of subwavelength ripples. Instead of easy production and observation of the NSRs, DSRs are only rarely found in ultra-short laser pulse (duration less than 100 picoseconds) irradiation of some selected materials, including fused silica [16], silicon [8,10,17], graphite [11] and some other materials [10,18,19]. Diverse mechanisms are proposed for explanation of the origins of DSRs, such as second and higher harmonic generation [10,20], SPPs [11] and self-organized effect [14,21].

The relationship between the formation mechanisms of NSRs and DSRs still remains a puzzle. The interference model of incident and surface scattered laser beam can effectively explain the correlation between the spatial period of NSRs and the incident angle, wavelength of laser irradiation, but not fit the period of the DSRs. The SPPs model excited from the rough surface by scattering with local defects is doubted as its intensity distribution is not at regularity but intricacy [22]. Rudenko A. gave a reasonable opinion that the surface scatter electromagnetic waves have been demonstrated to consist of the SPP, an evanescent cylindrical wave, and a Norton wave [23]. The DSRs models like second and higher harmonic generation [10] and grating-assisted SP-laser coupling [3,10] are not compatible with the spatial period of NSRs. The self-organized theory based on the ion sputtering and instability of thin liquid films is theoretically compatible with various ripple periods for any NSRs and DSRs, which attribute the directional asymmetry of laser-induced ripples to a spatial asymmetry of the excited-electron kinetic energies [21]. But it can only meet with the subwavelength periodic ripples that are perpendicular to the incident laser electric polarization. This does not agree with the scenario of ripples’ orientation parallel with laser polarization that is observed in dielectric materials [24], metals and semiconductor materials [10,25,26].

Another interesting subject of debate is whether the hierarchical periodic NSRs and DSRs can be constructed simultaneously on the same laser-irradiated area, which should be helpful to reveal the connection between the two, and to discover a unified theoretical model. Rare reports took notice of the mechanism and regularity of hierarchical periodic NSRs and DSRs formation. Yao et al. [27] used femtosecond laser scanning to fabricate large area subwavelength ripples on stainless-steel surfaces for a reinforced antireflection property. The induced ripples included both NSRs and DSRs, but the DSRs were not noted by the authors since the DSRs were not clear. Ji et al. [28] reported cross-periodic structures (NSRs and DSRs) on the silicon surface that the DSRs induced by a low laser pulse repetition rate of 1 kHz were not distinct and the formation mechanism remains in doubt. In contrast with the fabrication of NSRs, the DSRs usually need a higher repetition rate of the ultrashort laser pulse (above 76 MHz) [10] or an underwater environment [29,30]. Ahsan et al. [31] and Romano et al. [32] reported that the laser irradiation dose and the transient process had a significant influence on the ripple formation, suitable adjustment of the ultrafast laser-induced surface subwavelength ripples could offer a variety of colors. Gurevich et al. [33] proposed that the ripples formation cannot be explained in the frames of pure plasmonic theory and hydrodynamic instabilities are an important influence in the ripple formation. Liao et al. [34] reported that excitation of standing plasma waves at the interfaces between areas modified and unmodified by the femtosecond laser irradiation plays a crucial role in promoting the growth of periodic nanoripples. In fact, there are many diverse mechanisms proposed for the origins of laser-induced DSRs and NSRs, such as the second and higher harmonic generation [10,20], SPPs [11] and self-organized effect [14,21], so far no model can satisfy all the phenomena observed. Hence, the fabrication of in situ hierarchical periodic NSRs and DSRs remains a challenge when exploring their physical mechanisms and potential applications.

In this work, we present a picosecond (ps) laser induced hierarchical periodic NSRs and DSRs on stainless-steel surfaces. The evolution of DSRs to NSRs with laser peak power density deposition was observed, and the lattice orientation as the intrinsic characteristic of the stainless-steel material shows a significant role in the evolutionary process of DSRs to NSRs. A qualitative explanation of the ps laser-induced hierarchical periodic DSR and NSR formation mechanism is discussed and compared with a self-organized effect and the grating-assisted SP-laser coupling theory.

## 2. Materials and Methods

Stainless steel is one of the most extensively studied metals because of its versatility in industry and daily life. It has been reported that periodic NSRs are induced by the ultrashort pulse laser irradiation on stainless steel surface [35], but so far no DSRs. AISI 316L stainless steel sheets with dimensions of 50 mm × 50 mm and 1 mm of thickness were used in the experiment. Samples were mechanically polished and divided into two groups A and B. The group A samples were directly subject to the laser irradiation and the group B samples were firstly corroded by the surface erosion agent (HF 20 %vol. + HNO_3_ 10 %vol. + H_2_O 70 %vol.) before the subsequent laser irradiation. After the corrosion, the group B samples revealed the metallographic lattice structure on the surface. Before the laser irradiation, all the samples were cleaned by ultrasonic ethanol bath for 10 min. The same laser processing parameters were used for the fabrication of the hierarchical periodic NSRs and DSRs on the non-corroded (group A) and corroded (group B) sample surfaces.

**Figure 1:**
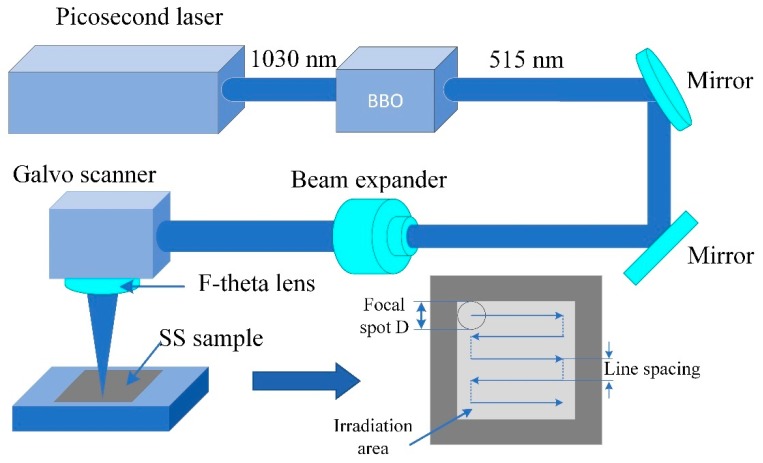
Schematic setup of the picosecond (ps) laser scanning system for fabrication of hierarchical periodic near-subwavelength ripples (NSRs) and deep-subwavelength ripples (DSRs).

The schematic setup of the experimental ps laser-scanning system is shown in Figure 1. The as-prepared samples were irradiated with a linearly polarized ps laser (Trumicro5050, TRUMPF Company, Ditzingen, Germany) with the repetition rate of 400 kHz and the pulse duration of 10 ps. The BBO (Beta barium borate, β-BaB2O4) crystal was utilized for frequency doubling of the incident laser beam from the wavelength of 1030 nm to 515 nm. Also, we set up the beam expander in order to achieve smaller focal spot size and higher laser fluence. A 2-D galvanometric scanner (SCANLab Company, Puchheim, Germany) with F-theta objective lens was used for the focused laser beam scanning in *x*-*y* directions. A *z*-direction stage was used to control the focal length of the laser beam spot since the laser beam was set perpendicular to the surface of samples. The focal spot diameter, defined as the gaussian-profile laser beam at 1/e^2^ of its maximum intensity, was approximately 16 μm. Different scanning speeds and power density were selected for fabrication of the hierarchical periodic dual subwavelength ripples. Large-area hierarchical periodic NSRs and DSRs were prepared by laser parallel line scanning on the sample surfaces.

The micro-morphologies of the prepared stainless-steel sample surfaces were investigated by laser-scanning confocal microscopy (OSL4100, OLYMPUS, Tokyo, Japan) and a scanning electron microscope (SEM, QUANTA 200F, FEI, Hillsboro, OR, USA), and the spatial period of the NSRs and DSRs were estimated from the high-magnification morphology images. Since there are reports that the single ripple line seems not always to be straight and perpendicular or parallel to laser polariton [36], which means the laser induced subwavelength ripples have bending and bifurcation, the orientation variation of ripple formation were also investigated.

## 3. Results and Discussion

### 3.1. Preparation of Hierarchical Periodic Near-Subwavelength Ripples (NSRs) and Deep-Subwavelength Ripples (DSRs)

During the interaction between pulsed laser and solid material, the cumulative history of receiving laser irradiation by a solid surface is essentially affected by the transient laser power density of single laser pulse (space factor) and the superposition rate of multiple laser pulses (time factor). Therefore, our experimental exploration was started by counted ps laser pulses stacking hitting a fixed point on the stainless-steel sample surface for investigation of the influences of space-time characteristics on laser-induced NSRs/DSRs, then expanded to line scanning for fabrication of large area hierarchical periodic NSRs and DSRs.

**Figure 2:**
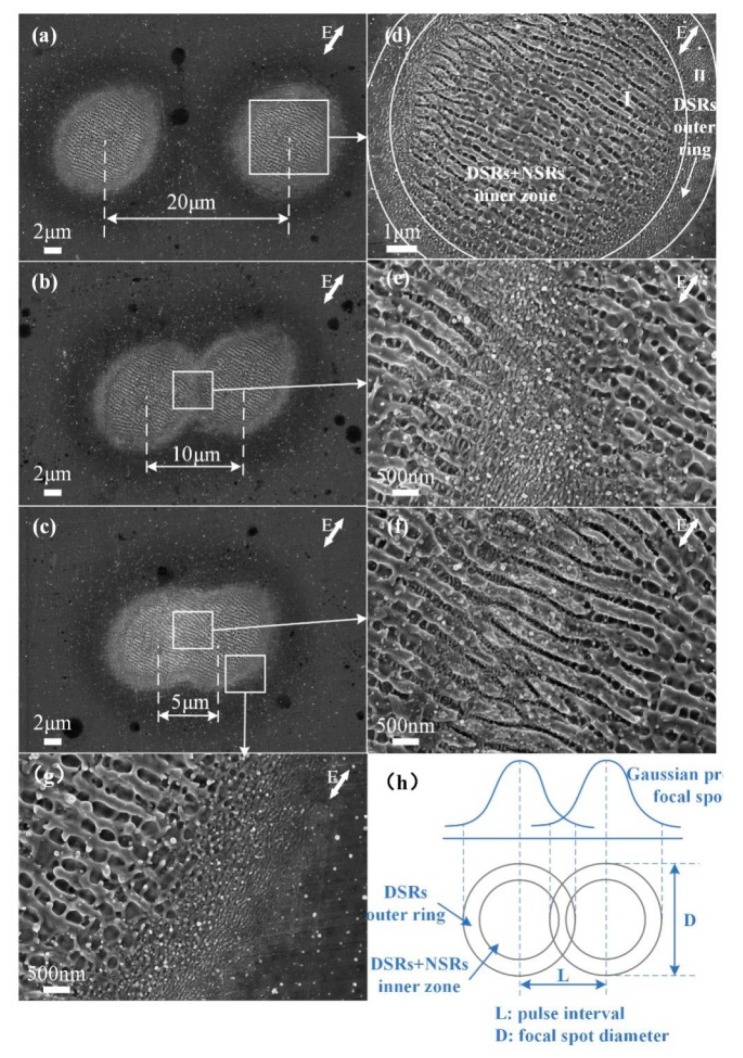
Scanning electron microscope (SEM) images of ps laser induced surface subwavelength ripples. Each of spot was carried out by 4 pulses with single pulse energy of 1.9 μJ and the hatch distance of the two spots was set as: (**a**) 20 μm, (**b**) 10 μm and (**c**) 5 μm. (**d**–**g**) The corresponding images of magnified areas separately. (**h**) The schematic of peak power density profile correlation with laser spots.

Figure 2 shows the SEM images of the ps laser induced surface subwavelength ripples at fixed points, each irradiated by 4 laser pulses with the single pulse energy of 1.9 μJ. The average laser power adopted in the experiment was 0.76 W. The schematic power density correlation of two adjacent ablation spots is illustrated in Figure 2h, where L is the center distance between two adjacent pulse points which equals 20 μm, 10 μm and 5 μm in Figure 2a–c, respectively. The laser power density *I(r)* at a certain point in the focal spot associating the material thermo-physical properties can be defined as the following equation:

(1) $$P=\overset{2\pi }{\underset{0}{\int }}\overset{+\infty }{\underset{0}{\int }}I_{0}e^{-2r^{2}/w_{0}^{2}}rdrd\theta =\pi w_{0}^{2}I_{0}/2$$

(2) $$I\left(r\right)=I_{0}\times e^{-2r^{2}/w_{0}^{2}}$$

where *p* = *E*/*t_p_* is the peak laser power, *E* is the single pulse energy, *t_p_* is the pulse duration, *I*_0_ is the maximum power density in the center of the focal spot and *w*_0_ is the waist radius of the laser beam.

The results clearly suggest the peak power density plays an important role in the formation of subwavelength ripples. Figure 2a shows the surface morphologies of two separate ablation craters with hatch distance *L* = 20 μm. The ablated crater presents a central, circular region with a radius of 8 μm can be characterized by two concentric rings, namely the dual DSRs + NSRs inner zone I and the single DSRs outer ring II, as shown in Figure 2d. In the inner zone I, the NSRs preferentially aligned perpendicular with the incident laser polarization (marked with the arrow *E*), which has a spatial period of 381 ± 23 nm as measured in the magnified Figure 2d. The DSRs situate in the valley of NSRs with orientation parallel to the incident laser polarization has a spatial period of 381 ± 23 nm. Considering the gaussian profile of the focal spot energy distribution, the laser peak power density threshold value of the inner zone I was calculated to be 91.9 GW/cm^2^. The ridges of the NSRs were steep and there were nano-particles on both the valleys and ridges. The outer ring II presents the characteristic texture of DSRs with a thickness of about 2 μm aligned along the laser polarization as shown in Figure 2g. The laser peak power density threshold value of the outer ring II with DSRs was calculated as 55 GW/cm^2^. As shown in Figure 2b, the hatch distance L is set to 10 μm and the overlapping rate is 33% (only the outer ring II of two ablated craters overlapped). In Figure 2e, it is clearly shown that more nanoparticles are dispersed on the DSRs and more DSRs formed in the valley of NSRs close to the overlapping area. With high laser peak power density in the center of the focal spot, the NSRs can be fabricated without pulse overlapping. Due to the low power density in the periphery of the focal spot, the DSRs formed without pulse overlapping while the NSRs formed with pulse overlapping. These phenomena are in accord with the reported theory that the threshold of multiple laser pulse material ablation is less than of single laser pulse ablation [37]. As shown in Figure 2c, the hatch distance L is set to 5 μm and the overlapping rate is 66%. The overlapping area consisted of the outer ring II and the inner zone I of the two ablated craters. A similar morphology can be seen in the non-overlapped area in Figure 2f, but the overlapping area of two adjacent craters were observed with the higher laser peak power density irradiation and the appearance of the hierarchical periodic NSRs and DSRs (in particular, the DSRs were vertically located in the valley of parallel NSRs). The ridge profile became coarse and the groove became narrow accompanied with the aggregation of plenty of nanoparticles on the ridge, and the coarsened ridge period changed from 201 nm to 249 nm at different locations. For applications, the evolutionary morphology from the periphery to the center of the crater due to the gaussian beam energy distribution suggests a precision control of the laser peak power density is necessary for fabrication of distinct hierarchical periodic NSRs and DSRs.

For the preparation of large-area hierarchical periodic NSRs and DSRs, it is worth exploring the relationship between the scanning speed and resultant morphology. The preparation of large-area hierarchical periodic NSRs and DSRs was implemented by a line-by-line laser scanning process. The spacing between the filled parallel lines was set to 10 μm. A series of laser scanning speeds from 50 mm/s to 1000 mm/s under the average laser power of 0.76 W were chosen to investigate the effect of space-time characteristic on large area laser-induced hierarchical periodic NSRs and DSRs. The numbers of overlapping laser pulses at any place of the laser scanning line path can be calculated by *N* = *D* * *f_p_*/*v*, where *D* = 16 μm is the diameter of focused Gaussian spot, *f_p_* = 400 kHz is the laser pulse repetition rate and *v* is the laser scanning speed as 50, 100, 300, 500, 800, 1000, 1300, and 1500 mm/s. Thus, the calculated overlapping pulse numbers (N) were 128, 64, 21, 13, 8, 6, 5 and 4, respectively.

**Figure 3:**
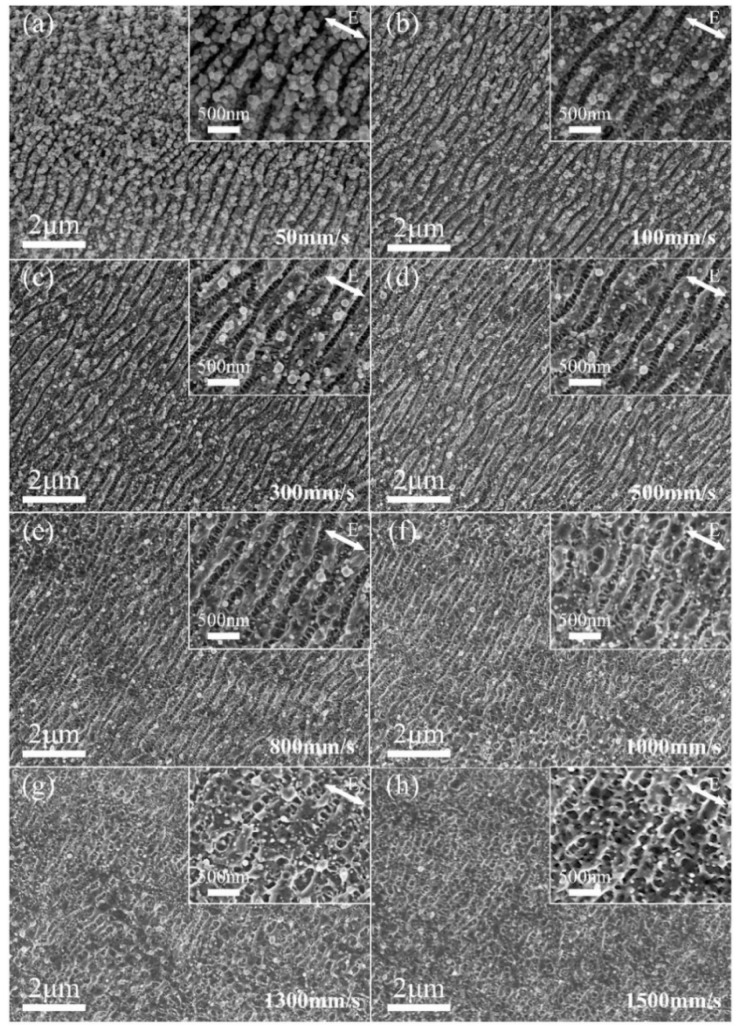
SEM images of subwavelength ripples prepared by different laser scanning speed and the fixed line spacing of 10 μm. The laser scanning speed of (**a**–**h**) is set to 50, 100, 300, 500, 800, 1000, 1300, and 1500 mm/s, respectively. The insets are zoomed views of the selected areas. The polarization of incident laser is indicated by the white double-headed arrow.

The SEM images of the prepared hierarchical periodic NSRs and DSRs with increasing laser scanning speed are shown in Figure 3. As shown in Figure 3a, there were multiple nanoparticles redeposited on the ridge of the NSRs and, and the NSRs nearly disappeared for the high power density pulse repeated irradiation under a relatively low scanning speed of 50 mm/s. The DSRs in the valley of the NSRs cannot be observed or have vanished due to the absorption of exceeded pulse energy irradiation. When the laser scanning speed was increased to 100 mm/s, the uniformity NSRs and DSRs could be captured easily from the zoomed view of Figure 3b. With the scanning speed increased up to 500 mm/s, the smooth and clean NSRs and rarely nanoparticles redeposition fabricated accompany with more regular DSRs formed in the valley of the NSRs. When the scanning speed was higher than 500 mm/s and lower than 1000 mm/s, the continuity of the DSRs and NSRs became worse and the bending of the DSRs was pronounced. When the scanning speed increased to 1300 mm/s and 1500 mm/s, the nanoripples formed were more blurred. It can be seen from Figure 3g,h that although the periodic ripples formed were not strictly along one direction, but the disorderly DSRs cross with NSRs could still be observed.

**Figure 4:**
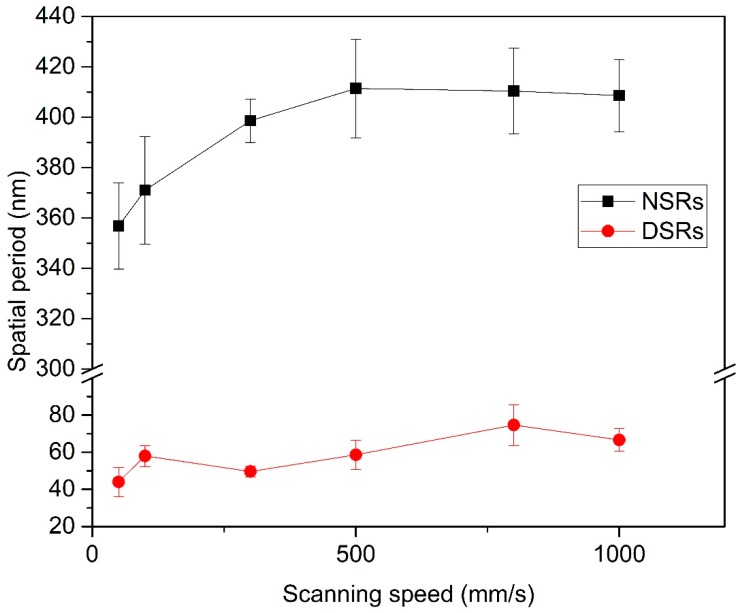
The spatial period of NSRs and DSRs irradiated at an average power of 0.76 W and various scanning speeds from 50 mm/s to 1000 mm/s.

As shown in Figure 4, the spatial period of the NSRs and the DSRs which irradiated at average power of 0.76 W and various scanning speeds from 50 mm/s to 1000 mm/s were measured. The influence of pulse energy overlapping in the charge of the scanning speed was investigated by means of the line and symbol chart. The spatial period of the NSRs shows a small increment from 356.8 ± 17 nm to 411.4 ± 19 nm as the scanning speed increased. This scenario is similar to the previous findings that the spatial period of the NSRs decreases with increasing pulse energy [8,35]. An efficient amount of high power density and its distribution which deposited on the pre-existing ripple could decrease the spatial period and the ridge of the ripple could become coarser for its inevitable pulse overlapping offset. When the scanning speed was higher than 500 mm/s, the spatial period reached a higher limit value of 411.4 ± 19 nm. We assume that the few pulses overlapping that of the low power density in the periphery of the follow-up pulse, interferes with the NSRs formation under that of high power density. Moreover, the DSRs in the valley formed this superimposed effect in chaos, too. The spatial period of the DSRs shows no obvious trend and fluctuates within a range of 44 nm to 74.6 nm.

**Figure 5:**
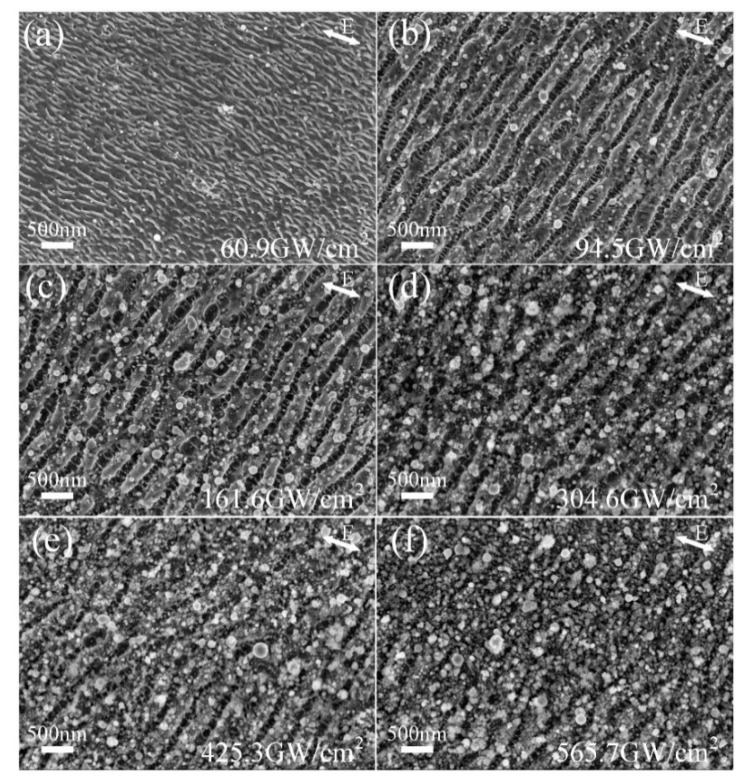
SEM image of the subwavelength ripples induced by different laser peak power density of (**a**) 60.9 GW/cm^2^, (**b**) 94.5 GW/cm^2^, (**c**) 161.6 GW/cm^2^, (**d**) 304.6 GW/cm^2^, (**e**) 425.3 GW/cm^2^ and (**f**) 565.7 GW/cm^2^, while the scanning speed is set to 500 mm/s and the line spacing is set to 10 μm.

Figure 5 shows the surface morphology evolution of ps laser-induced subwavelength ripples by increasing the average laser power from 0.49 W to 4.55 W at a fixed scanning speed of 500 mm/s, namely the peak power density increases from 60.9 GW/cm^2^ to 565.7 GW/cm^2^. As shown in Figure 5a, the surface is covered with uniform DSRs with orientation parallel to the incident laser polarization, since the peak power density is as low as 60.9 GW/cm^2^ (average laser power of 0.49 W). It is interesting that the DSRs induced at low peak power density are not straight lines in succession and are similar to dendrite or well-known hydrodynamic instabilities of thin liquid films [21]. By increasing the laser peak power density to 94.5 GW/cm^2^ (average laser power of 0.76 W), both the NSRs and DSRs were fabricated as illustrated in Figure 5b. More uniformity and regularity including continuous lines were observed on the samples when the laser peak power density was higher than 94.5 GW/cm^2^. In agreement with the previous reports, a correlation between the electric field polarization direction and the NSRs orientation was observed in all cases [3,10]. Due to the ultrashort pulse laser irradiation, the time of the pulse duration is not enough for the excited electron transfer energy to lattice and thermalization. All the nano-ripples were fabricated by the “cold” process with few heating effects, which is why so much high power density without molten pool but only more and more nano-particle aggregation.

As shown in Figure 5, the variation of laser peak power density has a great influence on the eventual morphology of the hierarchical periodic NSRs and DSRs. The spatial period is one of the most important topology parameters of the hierarchical periodic NSRs and DSRs, which may play a crucial role in its potential application such as superhydrophobicity, friction control and structural color.

**Figure 6:**
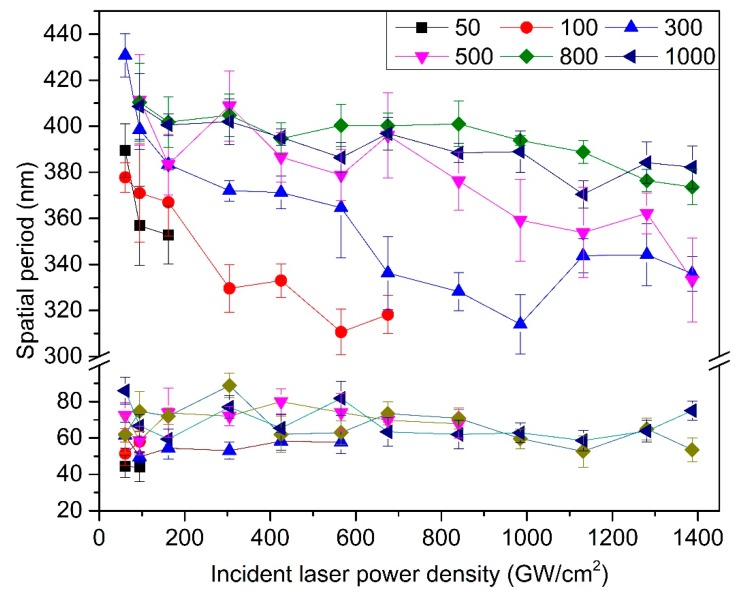
Comparison of the spatial period tendency of NSRs and DSRs with incident laser peak power density and scanning speed.

The spatial period tendencies of NSRs and DSRs with incident laser peak power density and scanning speed are shown in Figure 6. The spatial period of the NSRs decreases as the incident laser peak power density increases. Under the scanning speed of 50 mm/s, when the laser peak power density increases from 60.9 GW/cm^2^ to 161.6 GW/cm^2^, the period of the NSRs decreases from 389 ± 11 nm to 352 ± 12 nm. When the laser peak power density is greater than 161.6 GW/cm^2^, since the scanning speed is relatively low, the much greater pulse overlap results in the preformed periodic ripples’ disappearance. A similar scenario can be observed when the scanning speed is 100 mm/s and the laser peak power density greater than 675.2 GW/cm^2^. Moreover, the spatial period of the NSRs increases as the scanning speed increases, which agrees with the previous section. When the scanning speed is between 300 mm/s and 1000 mm/s, the NSRs were fabricated on the surface of the sample, and the period of the NSRs decreases from 430 ± 10 nm to 382 ± 14 nm with the laser peak power density increase.

On the other hand, the period of DSRs fluctuates at 65 ± 21 nm, and there is no obvious trend with the incident laser peak power density and scanning speed. The formation of the DSRs precedes the NSRs when the incident laser power density is slightly larger than the ablation threshold of the material. The periodic fluctuation is related to the local transient free electron density fluctuation of the material. The spatial period of the subwavelength ripples fabricated at different laser peak power density share a similar tendency with the scanning speed, which means the power density of incident laser has a decisive role in the light–matter interaction.

**Figure 7:**
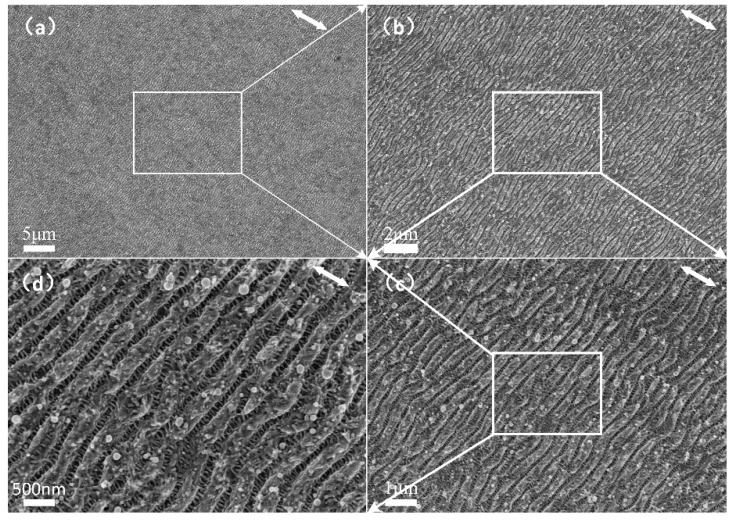
SEM micrographs of the prepared large-area hierarchical periodic NSRs and DSRs on the stainless-steel sample surface. (**a**–**d**) is the magnified picture of the selected area which indicated by a rectangular of white line.

Finally, a large area of the hierarchical periodic NSRs and DSRs was fabricated on the stainless-steel surfaces by a set of optimized laser-scanning parameters. The scanning speed of 500 mm/s and the laser average power of 0.76 W were chosen. Figure 7 shows the SEM micrographs of the prepared large area hierarchical periodic NSRs and DSRs. The spatial period of obtained NSRs and DSRs were 356 ± 17 nm and 58 ± 15 nm, respectively.

### 3.2. Lattice Orientation of Grains as a Factor in the ps Laser-Induced Hierarchical Periodic NSRs and DSRs

**Figure 8:**
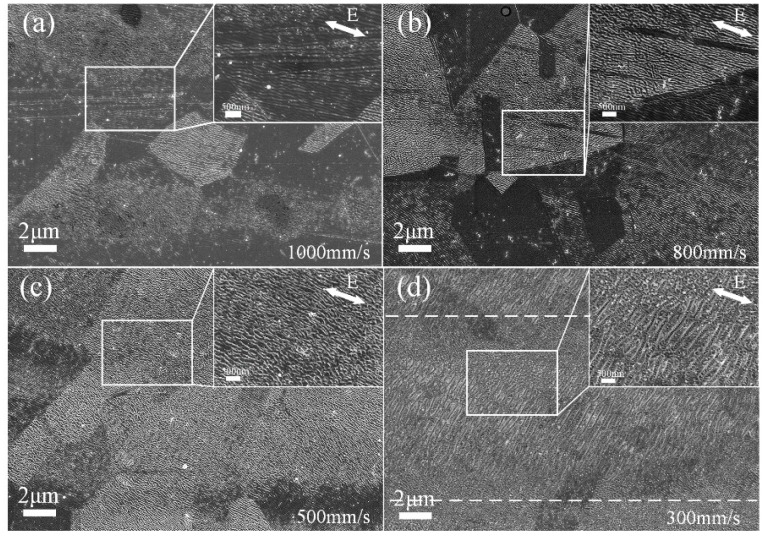
SEM images of subwavelength ripples prepared by different laser scanning speed and the fixed line spacing of 10 μm. The laser scanning speed is set to (**a**) 1000 mm/s, (**b**) 800 mm/s, (**c**) 500 mm/s, and (**d**) 300 mm/s, respectively. The insets are zoomed views of the selected areas. The polarization of the incident laser is indicated by the white double-headed arrow.

An interesting phenomenon has been found in that the lattice orientation of grains is a factor in the ps laser-induced hierarchical periodic NSRs and DSRs on the stainless-steel surfaces. The evolutionary differences of the subwavelength periodic ripples with decreasing laser scanning speed under low peak power density (60.9 GW/cm^2^, average laser power of 0.49 W) are revealed in Figure 8. By comparing Figure 8a,b we see that with the decrease of laser scanning speed (an increase of the overlapping pulse number N and laser energy absorption), the DSRs can be observed on stainless steel sample surface irradiated with all the different laser-scanning speeds (300~1000 mm/s), but the NSRs that are perpendicularly oriented only appear at low scanning speed (<300 mm/s, Figure 8d). With the high laser scanning speed of 1000 mm/s, the obscured DSRs were generated on the pristine surface and the spatial period of the nano-ripples was 58 nm. The ridges and valleys of the ripples are very smooth and these blurred DSRs show a regular arrangement that seems to be influenced by the lattice orientation of the material characteristics, as shown in Figure 8a,b. The initial DSRs formed at low peak power density have a conformance with the metallic grains, which has never been reported before. As shown in Figure 8a,b, the formation of DSRs has preferentially occurred in some “blocks”, which is clearly related to the lattice orientation of the material itself. When the laser-scanning speed is decreased to 500 mm/s, the DSRs became distinct and the area induced by the lattice orientation became unclear because the power density from the laser pulses is high enough for the valence electrons excitation upon reaching the material ablation threshold [37]. When the laser-scanning speed is lower than 300 mm/s, the hierarchical periodic NSRs and DSRs are uniformly generated, as shown in Figure 8d. But at the scanning line overlapping area of Figure 8d, which is similar to the Figure 2b scenario as the two adjacent DSRs outer rings overlapped, only DSRs are fabricated because the peak power density is lower than the threshold value of 91.9 GW/cm^2^.

**Figure 9:**
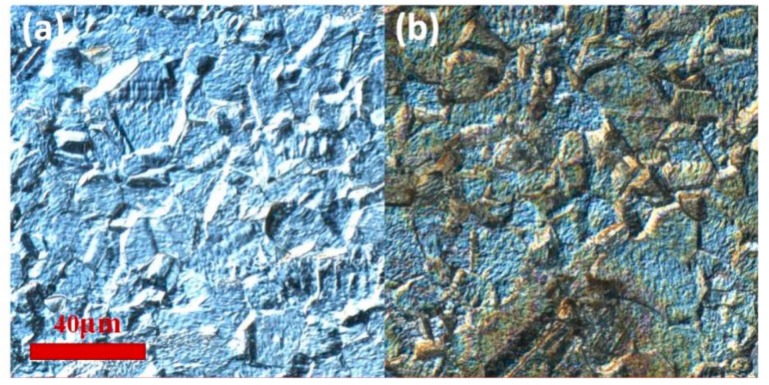
The metallographic lattice structure before and after the laser scanning treatment of 0.49 W and 1000 mm/s are shown in the confocal laser-scanning microscope images, (**a**): before, (**b**): after.

To further verify this hypothesis, we performed a pre-corrosion treatment on the stainless-steel samples in order to reveal the metallographic lattice structure on the surface. Hydrofluoric acid–nitric acid aqueous solution was selected on account of the grain of 304 stainless steel which is austenite grain. The metallographic lattice structure before and after the laser scanning treatment are shown in the confocal laser scanning microscope images of Figure 9a,b, respectively. Some grains turned a dark grey color after the ps laser irradiation, since the laser induced nano-scale ripples always present dark color in optical microscopes.

**Figure 10:**
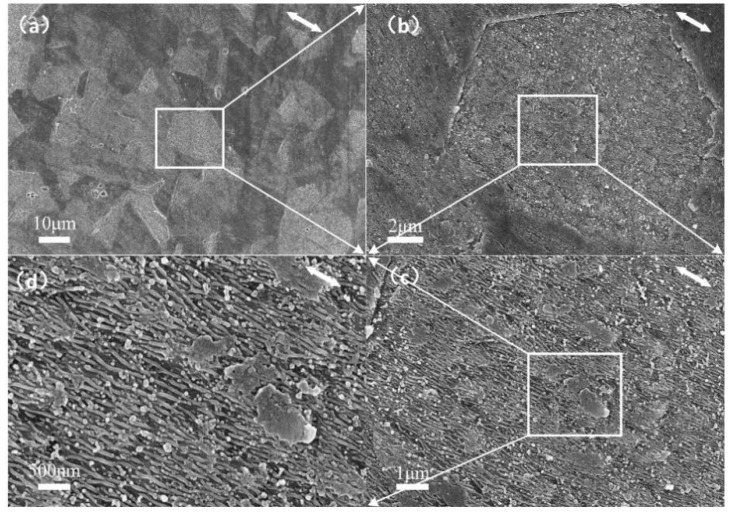
SEM images of the same sample of Figure 9b. (**a**–**d**) is the magnified picture of the selected area which indicated by a rectangular of white line.

Figure 10 shows the SEM micrograph of the same sample of Figure 9b, where the DSRs similar to those in Figure 8a–c can be observed, and the formation of DSRs is affected by the lattice orientation of stainless steel. On the different grains of the stainless-steel sample, the DSRs are selectively generated. The DSRs are found in adjacent grains with little differences in orientation, as shown in Figure 8a,b. The lattice orientation variations, which are the manifestation of grain atomic bulk density differences, are believed to have an intrinsic fluence on the initial formation of DSRs. Moreover, the DSRs formation conformance with the grain structures is sensitive to the peak power density irradiation of the material. In this study, the energy threshold of the lattice orientation and the atomic bulk density affects the DSRs’ formation. When the power density goes beyond the lattice binding energy of the stainless steel, the effect of the lattice on the initial formation behavior of DSRs disappears.

### 3.3. Mechanism for the Picosecond (ps) Laser-Induced Hierarchical Periodic NSRs and DSRs

According to the above discussed facts, a feasible mechanism model should be compatible with the three clues that are discussed above: (a) DSRs can be independently generated at a relatively low peak power density, but the generation of NSRs need much higher peak power density and accompanied by DSRs in the valleys of the NSRs. (b) The orientation of the periodic DSRs is parallel to the polarization of incident laser and perpendicular to the orientation of the periodic NSRs which coincides with the laser-induced SPPs. (c) The formation of the DSRs at low peak power density has a conformance with the metallic grain structures, and preferentially occurs in the interior of the grains that have low surface atomic planar densities. Neither the classical scattering wave theory nor the SPPs excitation theory can explain the formation of DSRs in which spatial period much smaller than the incident laser wavelength [11]. The shortcoming of the self-organization theory is the influence of asymmetric ionization kinetic energy affected by the polarization of the laser electric field, which cannot explain the hierarchy and polarization dependence of different ripples in this experiment [21]. Second-harmonic generation (SHG) is only found during the irradiation of certain materials, the present experiments cannot clearly show that SHG is involved in the formation of DSRs on any surface [38].

**Figure 11:**
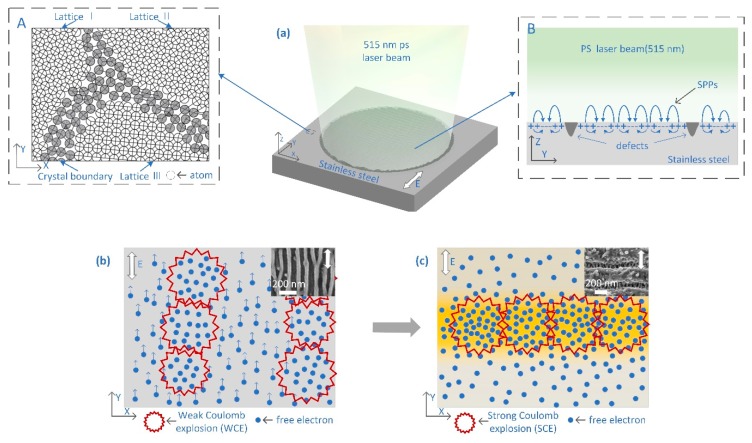
Schematic of the surface plasmon polariton (SPP)-modulated periodic coulomb explosion for the formation mechanism of hierarchical periodic NSRs and DSRs. (**a**) The picosecond laser irradiated on the stainless-steel surface. The dotted frame (**A**) indicated the atomic arrangement of the grains of 304 austenitic stainless steel. The dotted frame (**B**) shows the surface plasmon standing wave field induced at high energy density during laser irradiation. (**b**) The formation of DSRs under the material removal of coulomb explosion. (**c**) The NSRs induced by the periodically modulated by the surface plasmon standing wave.

A qualitative explanation based on SPP modulated periodic coulomb explosion is proposed for the formation mechanism of hierarchical periodic NSRs and DSRs. As shown in Figure 11, the atomic arrangement of the grains of 304 austenitic stainless steel is indicated in the dotted frame A. The white double-headed arrow shows the polarization of the incident laser. During a picosecond laser pulse duration, electron-absorbing photon ionization is always present, and the free electrons after ionization move in the direction of the electric field under the action of the laser electric field, forming a free electron gas limited to the surface movement. However, the concentration of free electrons on the surface of the sample is not uniformly distributed due to the surface roughness and defects, which lead to the initiation of the local non-thermal phase change coulomb explosion [39] at the place where local electron ionization intensity is large enough. As shown in Figure 11b, the material removal of coulomb explosion then makes the free electron gas movement blocked and concentrated at the adjacent place along the laser electric field, which results in the subsequent coulomb explosion chain that forms the DSRs. When the incident laser power density increases to a high threshold value, the laser electric field (Transverse Magnetic wave, TM wave) induced constraint SPPs will propagate along the metallic surface but perpendicular to the laser electric field orientation, and attenuate along the vertical direction of the metallic surface [40]. The dotted line frame B in Figure 11a shows the surface plasmon standing wave field induced at high energy density during laser irradiation. When the incident light is irradiated to a defect on the surface of the stainless steel, the SPPs are excited by the coupling of the surface electron plasma oscillation, and the surface plasmon standing wave is induced when the adjacent surface defects are separated by a certain distance.

Figure 11c shows that the surface energy field of the stainless-steel sample was being periodically modulated by the surface plasmon standing wave action, which made the free electron concentration increase and a strong coulomb explosion occurred. The subsequent free electrons under the SPP-modulated electric field are blocked and concentrated at the adjacent place along the SPPs’ propagation direction, which results in the subsequent coulomb explosion chain that forms the NSRs with orientation vertical to the laser polarization directions. The weak electron concentration in the valley after the strong coulomb explosion is likely to form a micro coulomb explosion, thereby forming the DSRs in the valley. For the inevitable pulse overlapping offset, when the peak power density is too high, the hierarchical periodic NSRs and DSRs structures will be partly destroyed and formed in chaos.

## 4. Conclusions

Although many notable works have been undertaken over the years to investigate ultrafast laser-induced periodic surface subwavelength ripples (NSRs and DSRs), how to simultaneously construct large-scale hierarchical periodic NSRs and DSRs still remains a challenge. The preparation regulation of the picosecond laser induced hierarchical periodic NSRs and DSRs on stainless-steel surfaces is investigated, and a unified qualitative explanation based on a SPP-modulated periodic coulomb explosion is proposed in this work. The main conclusions obtained are as follows:

(1) The peak power density of incident laser makes a decisive role in the light–matter interaction which leads to the generation of hierarchical periodic NSRs and DSRs. The DSRs can be independently generated at a relatively low peak power density, whereas the generation of NSRs needs much higher peak power density and are accompanied by DSRs in the valleys of the NSRs. The orientation of the periodic DSRs is perpendicular to that of the periodic NSRs, which was always found to be perpendicular to the polarization of the incident laser and coincides with the laser-induced SPPs.
(2) The formation of the DSRs at low peak power density has a conformance with the metallic grain structures, and preferentially occurs in the interior of the grains that have low surface atomic planar densities. Moreover, the spatial period of the NSRs is determined by the peak power density absorption and the material intrinsic thermo-physical properties.
(3) A qualitative explanation based on SPP-modulated periodic coulomb explosion is proposed for the formation mechanism of hierarchical periodic NSRs and DSRs. During a picosecond laser pulse duration, the photon-absorbed free electrons’ motion initiates the locally non-thermal phase change coulomb explosion and results in the subsequent coulomb explosion chain that forms the DSRs. The laser electric field (TM wave)-induced constraint on SPPs’ propagation along the metallic surface makes the free electron concentration increase and a strong coulomb explosion chain occurs that forms the NSRs with orientation vertical to the laser polarization direction.
(4) The preparation of large-area hierarchical periodic NSRs and DSRs was implemented by a line-by-line laser scanning process within which either the incident laser power or scanning speed can be used as the control variable. The spatial periods of the obtained NSRs and DSRs were 356 ± 17 nm and 58 ± 15 nm, respectively.
(5) Further theoretical calculation and simulation are needed to verify the qualitative explanation and find routes to improve continuity and strict directionality of the hierarchical periodic NSRs and DSRs. The potential applications such as wettability, tribology, structural color are promising since the laser-scanning preparation method is simple and precisely controllable.
